# Hydride Formation in Single Palladium and Magnesium Nanoparticles Studied By Nanoplasmonic Dark-Field Scattering Spectroscopy

**DOI:** 10.1002/adma.201101976

**Published:** 2011-09-05

**Authors:** Timur Shegai, Christoph Langhammer

**Affiliations:** Division of Bionanophotonics, Department of Applied Physics, Chalmers University of Technology412 96 Göteborg, Sweden

Nanosystems can exhibit interesting novel chemical and physical properties and are widely exploited for their functionalities.^1^–^6^ Scrutinizing such systems and relating, e.g., details in their chemistry and geometry to functionality, remains, however, a significant experimental challenge. Unwanted artifacts and averaged responses are always present in ensembles of a sample material and are mainly caused by inhomogeneous size distributions and differences in the local chemistry and the local structure of (quasi-identical) nanoentities in the ensemble. Experiments aiming at the characterization of single functional nanoentities have the potential to completely eliminate such problems, which facilitates highly relevant systematic studies of correlations between details in nanoentity geometrical, physical and chemical properties, and functionality. In applications such as catalysis or other areas of gas–solid or liquid–solid interactions as well as in solid-state reactions issues including temperature or mass-transport gradients present in powder samples, which often have to be stabilized by surfactants or similar, can cause significant problems in data interpretation and quantification by causing false apparent kinetics, “smeared out” phase transitions, etc. The same problems are also significant in situations when statistical averaging over even (potentially) identical particles may hide interesting phenomena such as oscillatory kinetics.^7^ In general, such effects make it very difficult or even impossible to correlate details in the local structure, size, and chemical composition of the probed nanomaterials with their functionality, e.g., kinetics, phase transitions, thermodynamics, catalytic activity, etc., when ensemble samples are used.

In the field of nanoplasmonic sensing, the introduction of dark-field scattering spectroscopy (DFSS) has made it possible to study the optical properties of single nanoparticles and to study effects of particle size and particle shape^8^ as well as the refractive index of the surrounding medium^9^ on the localized surface plasmon resonance (LSPR) in single Au and Ag nanoparticles. It has also been shown that it is feasible to directly observe a chemical redox reaction on the surface of a single Au nanocrystal^10^ or to utilize the LSPR in a single Au nanoparticle to probe the insulator–metal phase transition in an adjacent VO_2_ film.^11^ However, so far these DFSS studies have been limited to probe either adjacent film materials or, when probing particles, to Au or Ag systems and to particle sizes *D* > 30–50 nm, because Rayleigh scattering is proportional to *D*^6^, indicating that small particles do not scatter light efficiently enough to be detected with current charge-coupled device (CCD) detectors. Thus, plasmonic nanoparticles made from other metals, which would be of interest because of their functionality but which typically feature larger imaginary dielectric functions in the visible, such as Cu^12^ or many catalytically active transition metals such as Pt and Pd,^13^ have not been investigated by DFSS due to the predominantly absorptive nature of their LSPR, in particular in the small (*D* < 100 nm) particle size range. The above is very unfortunate in the sense that many functional nanosystems either are non-metallic and thus do not exhibit LSPR or consist of metals featuring highly absorptive LSPRs and are only of interest and functional at sizes *D* < 30 nm. For example, Au^3^ and other catalysts are typically on the order of 1–10 nm in size.

Here, we report following the indirect nanoplasmonic sensing (INPS)^14^–^16^ concept, a novel sensor–probed nanoparticle geometry, i.e., truncated Au nanocones with functionalized tips, fabricated in one physical vapor deposition (PVD) step using the self-assembly-based hole-mask colloidal lithography (HCL)^17^ method. The structures can be used for single-particle DFSS measurements on nanoparticles that are <30 nm and consist of absorptive metals or even dielectric materials, which themselves do not support LSPR. The key features that make INPS a very powerful experimental platform to study physical and chemical processes in and on functional nanosystems in situ under realistic application conditions demonstrated earlier are retained, even in single particle experiments.

We illustrate this by measuring hydride formation isotherms of single Pd and Mg nanoparticles <30 nm. Pd (and Pd hydride, PdH*_x_*) is a strongly absorptive plasmonic system while Mg hydride (MgH*_x_*) is an insulator that does not exhibit LSPR. Furthermore, Mg is, among the various light metals that could be used to store hydrogen as a hydride, one of the most promising candidates because of its low cost, ready availability, abundance, and low toxicity. The potential benefits of nanosizing Mg-based hydrogen storage systems thus make these results very relevant.^1^ As a second aspect of the experiments we specifically address the long-standing question of the origin of slope on the equilibrium plateau seen for hydride formation in small nanoparticles.^18^

For these experiments truncated Au nanocones, were fabricated using HCL. They featured a tip that was, according to the general INPS scheme, functionalized by a spacer layer located between the truncated Au nanocone and the functional nanoparticle at the tip of the entire structure (**Figure 1**a,d). Using this specific nanoarchitecture in a DFSS experiment, the truncated Au nanocone acts as plasmonic sensor and strong scatterer. Because its size is >>50 nm, its scattering signal will be easily detectable with state-of-the-art CCD detectors. It probes, through its enhanced plasmonic field, the adjacent functional but not necessarily plasmonic and/or scattering nanoparticle deposited onto the spacer layer on the tip of the cone structure. From a more general nanoplasmonic sensing point of view these nanocones have the advantage of strong near-field enhancement in the tip region, which provides high sensitivity, when partly excited along the out-of-plane axis, as is typically the case in DFSS.^9^

**Figure 1:**
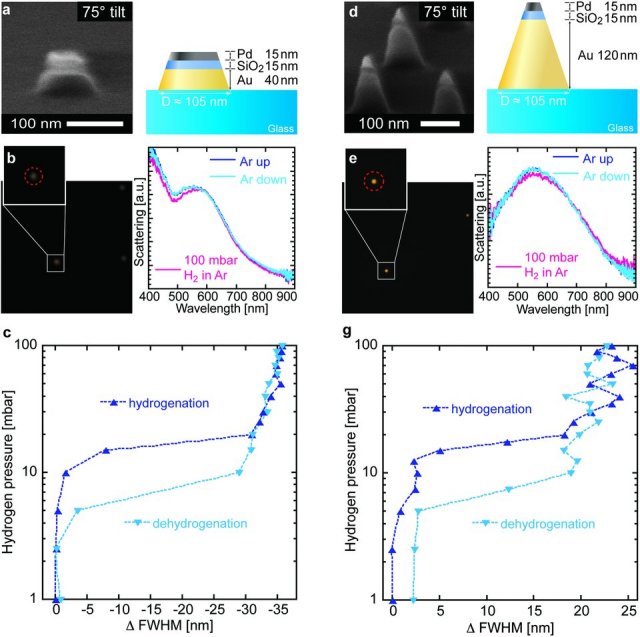
a) ESEM image and schematic depiction of an approximately 40 nm high truncated Au nanocone with a nominal base diameter of 105 nm, covered by a 15 nm SiO_2_ spacer layer and a Pd particle with approximate dimensions of 75 nm × 15 nm on the tip. b) DFS image of a single truncated 40 nm Au/SiO_2_/Pd nanocone (left) and its spectral response to subsequent exposure to 100% Ar, 10% H_2_ in Ar, and then 100% Ar (right). c) Corresponding optical hydrogenation isotherm at 23 °C. d) ESEM image and schematic depiction of an approximately 120 nm high truncated Au nanocone with a 15 nm SiO_2_ spacer layer and a Pd particle with approximate dimensions of 30 nm × 15 nm on the tip. e) DFS image of a single nanocone (left) and its spectral response to subsequent exposure to 100% Ar, 10% H_2_ in Ar, and then 100% Ar (right). f) Corresponding optical isotherm of the hydrogenation at 23 °C.

The fabrication of such conical structures is achieved by exploiting the typical-for-HCL successive decrease in diameter of the nanoholes in the fabrication mask upon material deposition. The latter effect is a result of material deposition onto the rims of the nanoholes in the mask and the subsequent shrinkage of the latter, which yields a corresponding decrease in the diameter of the growing nanoparticle during evaporation, thus resulting in conical structures. This fabrication approach conveniently facilitates the deposition of nanoparticles of interest directly on the tip of the Au cone sensor during the fabrication of the entire structure in one step with high flexibility in terms of material selection and combinations. Furthermore, by fine-tuning the fabrication parameters, i.e., the amount and thickness of the deposited tip materials and total height of the structure, the size of the active nanoparticle sitting on the tip of the Au nanocone sensor can be controlled very accurately and more complex structures featuring multiple layers of different materials are easily achieved, as illustrated below. We note that other lithography-based nanofabrication techniques such as electron-beam lithography could also be used to exploit the above effect.

Figure 1a shows a tilted environmental scanning electron microscope (ESEM, FEI Quanta 200 ESEM FEG, 20 keV) image (left) and a schematic depiction (right) of the typical nanoarchitecture of the first type of INPS nanocone structure studied by single-particle DFSS. It consists of an approximately 40 nm high truncated Au nanocone with a nominal base diameter of 105 nm, covered by a 15 nm SiO_2_ spacer layer onto which a 15 nm thick Pd particle was deposited. The latter has a diameter of ≍75 nm as can be seen in the ESEM image. Figure 1b shows a dark-field scattering (DFS) microscopy image of a single truncated Au/SiO_2_/Pd nanocone and its spectral response from subsequent exposure to 100% Ar, 10% H_2_ in Ar, and then 100% Ar (right). A very clear and reversible shift of the LSPR peak in the scattering spectrum, which is attributed to the LSPR in the stacked nanocone with the dominant contribution being from the Au base, induced by the exposure to hydrogen and the subsequent transformation of the Pd particle on the tip of the nanocone into PdH_*x*_ is seen. The LSPR shift is caused by the significantly different dielectric properties of the Pd and PdH*_x_* phases.^19^

Figure 1c shows the phase diagram or optical isotherm of hydrogen in a single Pd nanoparticle at 23 °C obtained by reading out the change in the full width at half-maximum (ΔFWHM) of the LSPR peak of the Au nanocone structure as a function of hydrogen partial pressure *p*. Previous experimental and theoretical considerations of LSPR-based measurements, as well as other optical approaches such as hydrogenography of thin films,^20^ of hydride formation in Pd nanosystems yielded a clear linear scaling of the LSPR signal (ΔFWHM) with the hydrogen concentration in the Pd nanoparticles.^15^, ^21^, ^22^ Therefore we will assume linearity even in these single particle experiments.

The *p*–ΔFWHM isotherms for hydrogenation and dehydrogenation show distinct *α*- (hydrogen in solid solution), *α*+*β* (plateau, mixed phase), and *β*-phase (hydride) regions as is characteristic for metal–hydrogen systems in general, and the PdH_*x*_ system in particular. The formation of a hydride phase in metals occurs at a certain concentration, the onset of the plateau, of dissolved hydrogen atoms due to attractive interactions between them.

Figure 1d–f summarizes an identical experiment as described above but for an arrangement yielding a significantly smaller probed Pd nanoparticle on the tip of a truncated Au nanocone. This was achieved by increasing the height of the truncated Au nanocone sensor to ≍120 nm. The tip of the sensor was functionalized, as before, with a 15 nm thick SiO_2_ spacer layer onto which 15 nm Pd was deposited (Figure 1d), yielding a Pd nanoparticle with a base diameter of only ≍30 nm.

Figure 1e shows a DFS image of a single 120 nm Au/SiO_2_/Pd nanocone (left) and its spectral response to subsequent exposure to 100% Ar, 10% H_2_ in Ar, and then 100% Ar (right). Again, also for the significantly smaller Pd particle in this experiment, a clear and reversible shift of the LSPR peak in the single-particle scattering spectrum induced by the formation of the PdH*_x_* phase can be observed. Figure 1f shows the single particle INPS *p*–ΔFWHM isotherms for hydrogenation and dehydrogenation. Before further interpretation and quantification of these data, a short comment on the crystallinity of these Pd nanoparticles is required. From earlier work we know that Pd nanodisks fabricated by using the same PVD system under identical conditions are polycrystalline with an average grain size on the order of 20 nm.^21^ We therefore expect a very similar polycrystalline microstructure even for the single Pd nanoparticles addressed here, at least for the large (75 nm × 15 nm) ones. The situation for the smaller particles (30 nm × 15 nm) might be slightly more uncertain because the particle size approaches the average grain size and questions related to the thermodynamic stability of, e.g., a grain boundary or even a dislocation, might become relevant. Nevertheless it is reasonable to assume that even the smaller Pd particles are polycrystalline.

We note the negative and positive ΔFWHM signals for the 40 nm and 120 nm high Au nanocone sensors, respectively. We speculate that the origin of the different signs of the shifts is related to the fact that in the first case (75 nm × 15 nm Pd) the Pd particle in fact exhibits a plasmonic resonance of its own, significantly overlapping spectrally^13^ with the one in the Au nanocone due to comparable heights and diameters, yielding a hybridized plasmonic system.^23^ In particular, the formation of the hydride phase introduces a very significant damping and weakening of the LSPR of the Pd particle,^21^ which in turn significantly weakens the coupling between the LSPR of the Au and the Pd particle, respectively. Therefore, a narrowing of the predominantly Au LSPR peak is observed upon hydride formation. In the second case, however, the plasmonic resonances in the Au nanocone and the Pd particle are spectrally not overlapping^13^ in the same way, yielding, from a plasmonic point of view, a completely different system with different optical response. We speculate that, in this case, the sensing is of more pure dielectric nature, i.e., that the LSPR of the Au and Pd particles do not hybridize and that only the change of the dielectric function of the Pd during hydrogenation is detected, in analogy to a plasmonic biosensing experiment. Further investigations and modeling are required to understand this effect in detail. These are, however, beyond the scope of this communication.

Interestingly, for both Pd particle sizes an almost identical hysteresis between the hydrogen sorption and desorption branches as well as a significant slope of the two-phase coexistence plateau is observed. In the literature, the appearance of a slope in the mixed phase regime of hydrided nanoparticles is interpreted as a clear sign of significant deviation from bulk properties, whose origin still is debated. Usually it is argued that the slope is induced by a combination of lattice strain, which may be caused at interfaces towards other materials or also, for very small particles <5 nm, by surface tension, structural heterogeneity of the particle, i.e., defects, and polydisperse particle size distribution in the sample material.^24^–^27^ Due to the absence of particle size distribution effects in our single particle experiments and because the particles are too big to exhibit surface-tension-related effects, the slope in the plateau region of the isotherms can here, for the first time, experimentally be attributed to lattice strain effects, most probably induced at the SiO_2_/Pd interface^28^ and, to a minor extent, to structural defects (discussion follows). This is illustrated in **Figure 2**c, which shows a 30 °C *p*-Concentration (*C*) isotherm for an ensemble of polycrystalline bulk-like (diameter *D* = 290 nm and height *h* = 60 nm) and structurally relaxed, by gentle thermal annealing and hydrogen cycling to release residual strain from the PVD process and potentially healing of some defects, Pd nanodisks measured by Zoric et al.^21^ and for an ensemble of identically annealed *D* = 50 and *h* = 15 nm Pd nanodisks. In the same figure a single Pd particle optical isotherm obtained from the 120 nm Au nanocone sensor structure, 30 nm × 15 nm Pd, not thermally annealed and measured after only few hydrogenation cycles, is shown for comparison. Here we also note that these three different structures were fabricated using the same PVD system with the same evaporation source and deposition conditions. The latter guarantees essentially identical microstructures (i.e. grain size, types, and density of defects) after deposition in all three cases. The lack of thermal anneal (annealing is difficult because it would even alter the Au sensor part of the nanocone structure) for the single particle case is expected to enhance the effects of interfacial lattice strain to a slopy plateau due to residual strain from the PVD process. Clearly, slope is absent in the ensemble data for the large disks and minor for the smaller disks, while the plateau of the single particle experiment has a significant slope. We attribute this observation i) to the larger relative weight of the Pd/SiO_2_ interface for 15 nm thick disks compared to the 60 nm case and ii) to an annealing effect reducing residual interfacial strain, with a smaller slope for the annealed ensemble 15 nm disks compared to the single particle case.

**Figure 2:**
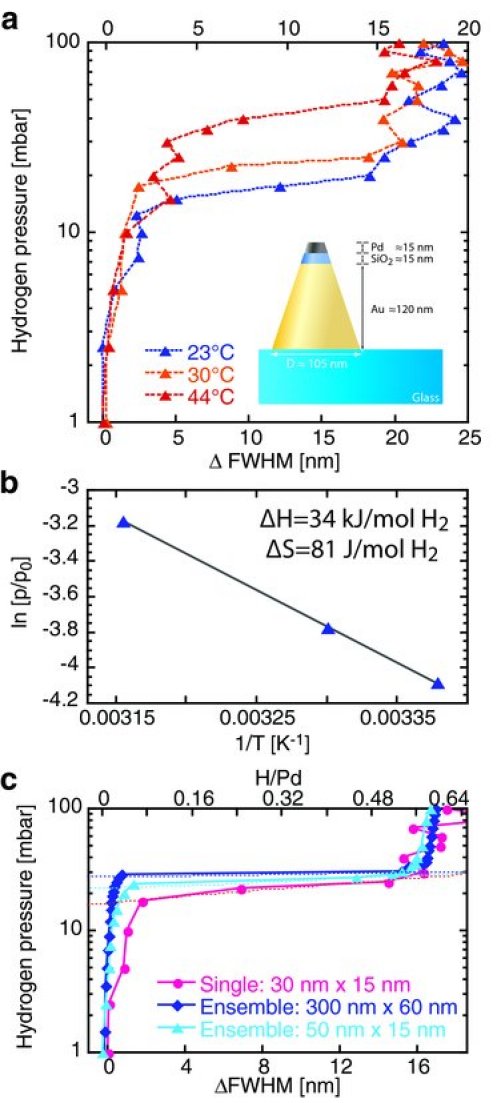
a) *p*–ΔFWHM isotherms at three different temperatures for a single 30 nm × 15 nm Pd nanoparticle. b) Corresponding Van't Hoff analysis yielding the enthalpy and entropy of hydride formation. c) *p*–*C* isotherm for an ensemble of structurally relaxed, by thermal annealing at 220 °C for 2 h, Pd nanodisks with mean diameter *D* = 290 nm and height *h* = 60 nm from^21^ (blue diamonds), an ensemble of thermally annealed (220 °C, 2 h) *D* = 50 nm and *h* = 15 nm Pd nanodisks (light blue triangles) together with an optical isotherm for a non-annealed single Pd nanoparticle (*D* = 30 nm, *h* = 15 nm, pink dots). Clearly, the plateau in the single particle experiment exhibits the largest slope. The primary *x*-axis belongs to the single particle experiment, the secondary one to the ensemble measurements.

To quantify the analysis of the hydride formation in a single Pd nanoparticle, we measured *p*–ΔFWHM isotherms at three different temperatures (Figure 2a) and a corresponding Van't Hoff analysis was carried out (Figure 2b), yielding the enthalpy and entropy of hydride formation. The obtained values indicate a destabilization of the hydride compared to typical bulk enthalpies.^29^

This observation agrees with the above-suggested interpretation of interfacial lattice strain being the main origin of the slopy plateau, i.e., that elastic constraints by the SiO_2_/Pd interface efficiently destabilize the hydride in analogy to reports by Baldi et al. for the Mg system.^28^ As main argument for this interpretation we suggest the fact that contributions from two other potential mechanisms are small, i.e., that the studied particles are too big to exhibit significant surface tension effects and that defects are not expected to alter the global (only possibly the local) thermodynamics of the system as long as they are not very abundant. Of course, minor contributions from defects may not be completely ruled out.

In **Figure 3**, we illustrate the flexibility of the HCL-fabricated nanocone single particle INPS approach by studying the formation of the hydride phase in a single Mg nanoparticle (size ≍35 nm × 15 nm) on a 120 nm high Au nanocone, sandwiched between two 5 nm thick Ti spacer layers and capped by a catalytic 10 nm thick Pd layer. In this particular nanoarchitecture, all the material layers have a specific function. As shown by Baldi et al.,^28^ the Ti layers elastically decouple the Mg particle during hydrogenation and prevent alloying between the Mg and Pd or Au. The Pd capping is necessary to prevent oxidation of the layers below and to catalyze hydrogen absorption in the Mg. This layered structure is clearly visible in the SEM picture (recorded on an ensemble for contrast reasons) in Figure 3a and schematically depicted in Figure 3b. In Figure 3c, the corresponding DFS image of a single truncated Au nanocone with the Ti/Mg/Ti/Pd functionalized tip is shown. The spectral response to subsequent exposure of the structure to 100% Ar, 2% H_2_ in Ar, and 20% H_2_ in Ar at a temperature *T* = 80 °C is summarized in Figure 3d. A clear shift of the scattering peak is observed. Figure 3e finally shows the optical isotherm of hydrogen in a single Mg nanoparticle at 80 °C, where the shift of the LSPR peak was analyzed as a function of hydrogen partial pressure. As for the Pd system above, the obtained isotherm shows distinct *α*-, *α*+*β*-, and *β*-phase regions with a distinct plateau. Notably, the latter is significantly flatter than the similar Pd nanoparticle isotherms discussed above, most probably due to weaker elastic constraints exhibited on the system by the Ti/Mg interface^28^ compared to the Pd/SiO_2_ system. We also note that a possible contribution to the scattering signal stemming from the hydrogenation of the Pd capping layer is negligible under the specific conditions of the experiment because the equilibrium pressure for the Pd/H system is of the order of 250 mbar at 80 °C, i.e., the Pd cap is still deep in the *α*-phase.^21^

**Figure 3:**
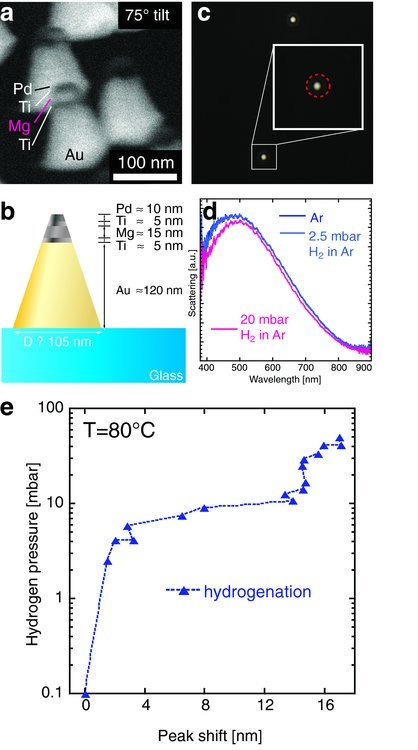
ESEM image (a) and schematic depiction (b) of 120 nm high truncated Au nanocones functionalized with a layered Ti/Mg/Ti/Pd tip. The Mg nanoparticle has a size of ≍35 nm × 15 nm. c) DFS image of a single Au nanocone with the Ti/Mg/Ti/Pd functionalized tip. d) Spectral response of a single nanocone architecture to subsequent exposure to 100% Ar, 2% H_2_ in Ar, and 20% H_2_ in Ar at atmospheric pressure and at *T* = 80 °C. e) Optical isotherm of hydrogen in a single Mg nanoparticle at 80 °C.

In conclusion, we have demonstrated, for the first time, how DFSS on single-particle nanoplasmonic sensors can be used to study, in detail and in real-time, the formation of a new phase in a single nanoparticle in situ by carrying out systematic studies of the hydride formation thermodynamics in single Pd and Mg nanoparticles. Notably, with these experiments, plasmonic DFSS measurements on single particles, which are <30 nm and can consist of absorptive plasmonic metals or are dielectric materials that themselves do not support LSPR, were demonstrated to be feasible. This was achieved by exploiting HCL-nanofabricated, single, truncated Au nanocones as nanoplasmonic sensors. The sensors were functionalized with the nanoparticle to be investigated by depositing material multilayers with distinct functions for each individual layer on the tip of the truncated Au nanocones during the same PVD deposition step as the Au nanocones. This renders the method highly efficient, flexible, and widely applicable.

In a more general perspective, due to the generic nature of the suggested experimental strategy, the above results are of broad interest because single particle studies of the kind reported here are of key importance for the development and understanding of efficient devices relying on functional nanomaterials, in particular when subtle differences in functional nanoparticle size, shape, crystallinity (e.g., exposed facets), or chemistry become important and may drastically affect the overall performance of the targeted system.

## Experimental Section

*Nanocone Fabrication*: The truncated nanocone structures were fabricated using HCL.^17^ As substrates, circular glass slides (Menzel Gläser) with a diameter of 25 mm and a thickness of 0.2 mm were used. The deposition of the nanocone materials for the Pd experiments was carried out in electron beam evaporator (AVAC HVC600) with a water-cooled sample holder at a base pressure of 2 × 10^−6^ mbar and an evaporation rate of 3 Å s^−1^ for Au, 0.5 Å s^−1^ for SiO_2_, and 1 Å s^−1^ for Pd. The deposition of the nanocone materials for the Mg experiments was carried out in an ultrahigh vacuum (UHV) electron beam evaporator (APX Scientific Instruments) at a base pressure of 2 × 10^−8^ mbar using an evaporation rate of 2 Å s^−1^ for Au, 1 Å s^−1^ for Ti, 0.5 Å s^−1^ for Mg, and 1 Å s^−1^ for Pd.

*DFS Spectroscopy*: The sample was mounted on an *XY* stage of a Nikon Eclipse TE2000-E inverted microscope, equipped with a Nikon 60x numerical aperture NA = 0.7 air objective. For dark-field imaging and spectroscopy a 0.8–0.95 air dark-field condenser (Nikon) was used. Dark-field spectra were recorded using a fiber-coupled spectrometer (Andor, SR-303I- B) and color images were recorded with Nikon D300s digital single-lens reflex (DSLR) camera.

*Hydrogen Storage Experiments*: The experiments were carried out in a heated stainless steel flow cell (volume approximately 110 μL), in which the sample was mounted as the optical window towards the microscope objective and a second fused silica window was mounted towards the dark-field condenser. A set of mass flow controllers (Bronckhorst low Δ*p*) with different working ranges was used to obtain the desired concentrations of hydrogen in the flow cell. The flow of the Ar carrier gas was adjusted after a change in hydrogen concentration by altering the hydrogen flow to keep the total gas flow constant at 100 mL min^−1^ during each measurement. The flow cell temperature was controlled by a glass fiber insulated kanthal heating wire connected to a Delta Elektronika SM-800 power supply.
